# Co-infection of TYLCV and ToCV increases cathepsin B and promotes ToCV transmission by *Bemisia tabaci* MED

**DOI:** 10.3389/fmicb.2023.1107038

**Published:** 2023-03-16

**Authors:** Ding-Yi-Hui Lu, Jin-Yu Liao, Anugerah Fajar, Jian-Bin Chen, Yan Wei, Zhan-Hong Zhang, Zhuo Zhang, Li-Min Zheng, Xin-Qiu Tan, Xu-Guo Zhou, Xiao-Bin Shi, Yong Liu, De-Yong Zhang

**Affiliations:** ^1^Subcollege of Longping, College of Biology, Graduate School of Hunan University, Changsha, China; ^2^Hunan Academy of Agricultural Sciences, Institute of Plant Protection, Changsha, China; ^3^Department of Entomology, University of Kentucky, Lexington, KY, United States; ^4^Research Center for Biomaterials, Indonesia Institute of Sciences, Cibinong, Indonesia; ^5^Institute of Vegetable, Hunan Academy of Agricultural Sciences, Changsha, China

**Keywords:** *Bemisia tabaci*, tomato chlorosis virus, tomato yellow leaf curl virus, single infection, co-infection

## Abstract

Tomato disease is an important disease affecting agricultural production, and the combined infection of tomato chlorosis virus (ToCV) and tomato yellow leaf curl virus (TYLCV) has gradually expanded in recent years, but no effective control method has been developed to date. Both viruses are transmitted by *Bemisia tabaci* Mediteranean (MED). Previously, we found that after *B. tabaci* MED was fed on ToCV-and TYLCV-infected plants, the transmission efficiency of ToCV was significantly higher than that on plants infected only with ToCV. Therefore, we hypothesize that co-infection could enhance the transmission rates of the virus. In this study, transcriptome sequencing was performed to compare the changes of related transcription factors in *B. tabaci* MED co-infected with ToCV and TYLCV and infected only with ToCV. Hence, transmission experiments were carried out using *B. tabaci* MED to clarify the role of cathepsin in virus transmission. The gene expression level and enzyme activity of cathepsin B (Cath B) in *B. tabaci* MED co-infected with ToCV and TYLCV increased compared with those under ToCV infection alone. After the decrease in cathepsin activity in *B. tabaci* MED or cathepsin B was silenced, its ability to acquire and transmit ToCV was significantly reduced. We verified the hypothesis that the relative expression of cathepsin B was reduced, which helped reduce ToCV transmission by *B. tabaci* MED. Therefore, it was speculated that cathepsin has profound research significance in the control of *B. tabaci* MED and the spread of viral diseases.

## 1. Introduction

Tomato chlorosis virus (ToCV) belongs to the genus *Crinivirus* in the family *Closteroviridae* (Accotto et al., 2001; Wintermantel and Wisler, 2006). It was first discovered in Florida in the United States in the mid-1990s and subsequently spread worldwide (Dalmon et al., 2005; Hirota et al., 2010; Fiallo-Olivé et al., 2011; Vargas et al., 2011; Arruabarrena et al., 2014). To date, ToCV is known to infect 25 species of plants across 7 families (Sun et al., 2016). Once present in the tomato field, ToCV infection in tomatoes can frequently reach 100% (Fiallo-Olivé and Navas-Castillo, 2019), with ToCV infection in early-stage tomato plants causing yield loss of up to 76% (Farina et al., 2019). Additionally, co-infections occur with viruses of different genera, such as members of the genus Begomovirus (TYLCV), genus Orthotospovirus (TSWV), and others (Garcia-eano et al., 2006; Alfaro-Fernandez et al., 2010). The co-infection has been known to cause crop yield reduction over a large area with serious economic losses to agricultural production (Ding et al., 2019).

TYLCV belongs to the genus *Begomovirus* in the family *Geminiviridae* (Fauquet et al., 2005), and it was first discovered in Israel (Cohen and Harpaz, 1964), and then gradually spread to the Middle East, Mediterranean coast, Africa, Asia, and other places (Cohen and Antignus, 1994; Czosnek and Laterrot, 1997; Polston et al., 1999; Ueda et al., 2005). The virus is characterized by its high virulence, which makes it highly prevalent in a field suffering from tomato virus regiments (Pan et al., 2012). It has a wide range of host plants and a strong adaptability, as indicated by its high frequency of gene variation (Papayiannis et al., 2011; Shirazi et al., 2014). These factors make it difficult to prevent and control TYLCV. TYLCV infection on tomato plants can reduce tomato production greatly, especially when a breakout occurs (Kanakala and Ghanim, 2016; Prasad et al., 2020).

*Bemisia tabaci*, an omnivorous insect belonging to Homoptera, Aleyrodidae (Shi et al., 2017), is a kind of herbivorous agricultural insect with piercing mouthparts that primarily consume tomato leaves (Laurence, 1962). *Bemisia tabaci* has become an important insect to monitor due to its role as a vector for viral disease. *Bemisia tabaci* transmits ToCV in a semi-persistent way (Wintermantel and Wisler, 2006; Fortes et al., 2020), while *B. tabaci* transmits TYLCV in a persistent way (Rubinstein and Czosnek, 1997; Yan et al., 2018). Other than that, these two viruses cannot be transmitted by sap friction (Gilbertson et al., 2015), which makes *B. tabaci* even more important as the gateway for virus infection on tomato plants.

Co-infection by two viruses on a single plant has been shown to increase virus load/titer and cause more severe symptoms to manifest on plants compared to individual viral infection as demonstrated by the study of the southern rice black-streaked dwarf virus (SRBSDV) and rice ragged stunt virus (RRSV) co-infection on rice plants (Li et al., 2014, 2017). Li et al. (2017) also suggested that higher virus titers in the infected rice plants can lead to higher virus acquisition efficiency by the insect vectors, such as white-backed planthopper and brown planthopper. Other examples from the co-infection study of potato leafroll virus (PLRV) and potato virus Y (PVY) on potato plants also demonstrated that *Myzus persicae* and *Macrosiphum euphorbiae* (Homoptera: Aphididae), which are the vectors for both of the potato disease viruses, have a more efficient transmission rate when feeding on the co-infected plants than on PVY-infected plants alone (Srinivasan and Alvarez, 2007). These results indicate that synergism can improve the transmission efficiency of insect vectors and enhance the pathogenicity of the virus (Murphy and Bowen, 2006; Malik et al., 2010). Thus, it was suspected that tomato plants co-infected with ToCV and TYCLV may enhance the transmission rates of ToCV.

Among the control strategies of whitefly-transmitted viruses, RNA interference (RNAi) is one of the most important virus management methods. RNAi is a phenomenon in which small non-coding RNA (sncRNA) produced by long double-stranded RNA (dsRNA) induces efficient and specific degradation of homologous mRNAs (Mallick and Ghosh, 2012). RNAi is mainly used to block gene expression at the post-transcriptional level, inhibit translation, or promote heterochromatin formation, resulting in the inability to synthesize proteins and “gene silencing” or reduced expression levels (Czech and Hannon, 2010). RNAi technology has been widely used in agricultural control, especially for insect gene function (Fire et al., 1998).

In this study, to find out the key factors that can affect the transmission of ToCV by *B. tabaci* MED, we sequenced the transcriptome of *B. tabaci* MED that fed for 48 h on tomato plants co-infected by ToCV and TYLCV and infected only with ToCV. We found several differential genes; among them, we found a high expression of the cathepsin B gene in *B. tabaci* MED that fed on co-infected plants, which promoted the transmission of ToCV. Cathepsin belongs to cysteine proteases, which have a conserved active three-dimensional pocket composed of histidine, asparagine, and cysteine residues (Gasteiger et al., 2003; Hu et al., 2014). Cathepsin is known to regulate the infection and transmission of the virus. For example, cathepsin B can inhibit the acquisition of PLRV by aphids (Pinheiro et al., 2017).

We hypothesized that the relative expression of cathepsin B would be reduced after silencing cathepsin B, which would reduce ToCV transmission by *B. tabaci* MED. Several experiments were conducted to investigate the effects of cathepsin B on the transmission of ToCV in *B. tabaci* MED infected by co-infection and single infection. They are (1) comparing the ToCV accumulation in *B. tabaci* MED between infection groups; (2) comparing the results of the differentially expressed genes from transcriptome sequencing of *B. tabaci* MED between infection groups to detect changes of related transcription factors in *B. tabaci* MED; (3) comparing the relative expression and enzyme activity of cathepsin B in *B. tabaci* MED between infection groups; and (4) determining the virus acquisition and transmission efficiency of *B. tabaci* MED between infection groups after treatment with a cathepsin activity inhibitor and silencing cathepsin B. Our results will enable us to further understand the mechanism of virus transmission affected by the co-infection of ToCV and TYLCV and to prevent virus transmission effectively.

## 2. Results

### 2.1. Plant infection confirmation

**Figure 1:**
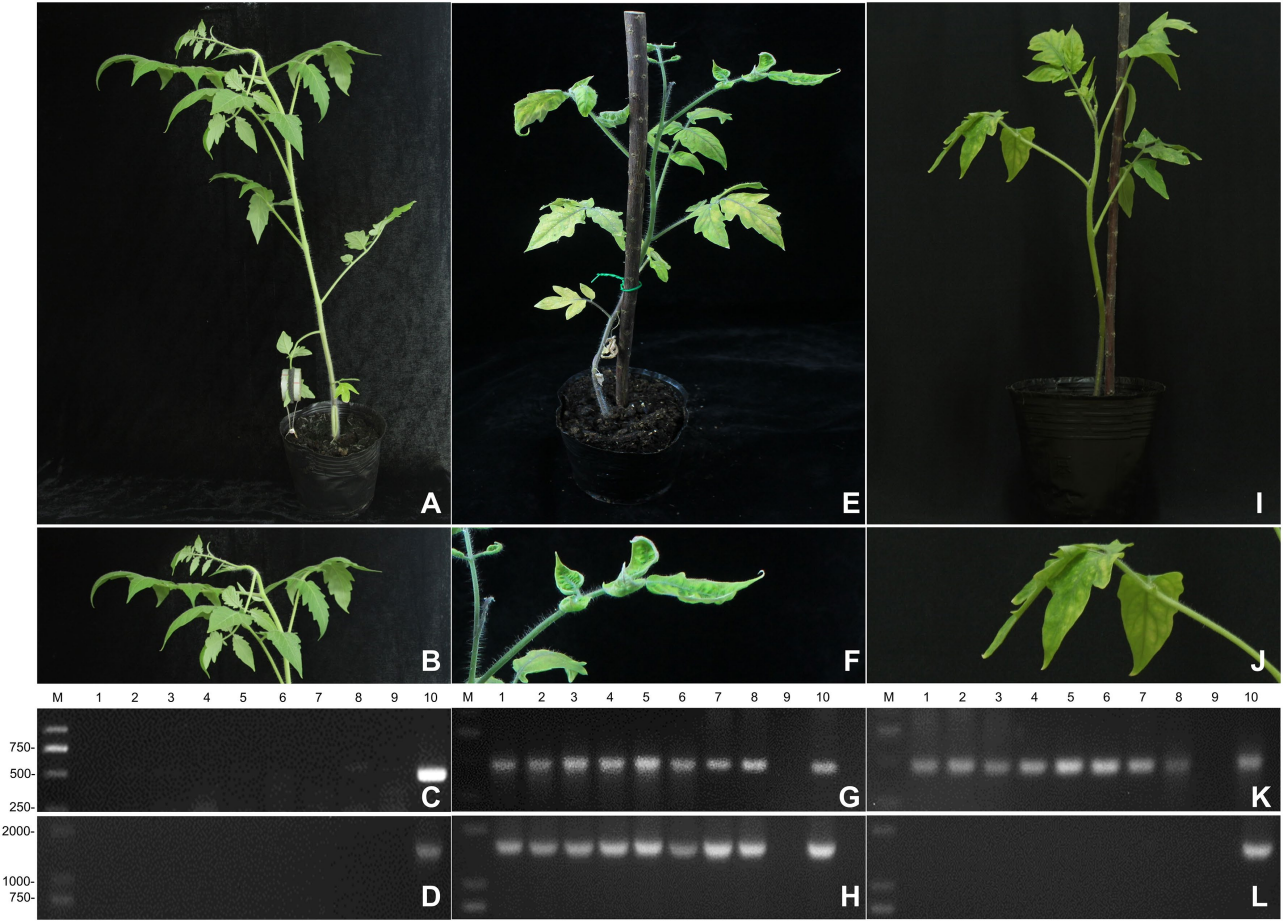
The infection of plants. **(A)** The symptoms of non-infected tomato plants. **(B)** Detailed symptoms of non-infected tomato plants. **(C)** Detection of ToCV in non-infected tomato plants by RT-PCR. **(D)** Detection of TYLCV in non-infected tomato plants by RT-PCR. **(E)** The symptoms of co-infected tomato plants. **(F)** Detailed symptoms of co-infected tomato plants. **(G)** Detection of ToCV in co-infected tomato plants by RT-PCR. **(H)** Detection of TYLCV in co-infected tomato plants by RT-PCR. **(I)** The symptoms of single-infected tomato plants. **(J)** Detailed symptoms of single-infected tomato plants. **(K)** Detection of ToCV in single-infected tomato plants by RT-PCR. **(L)** Detection of TYLCV in single-infected tomato plants by RT-PCR. M: DNA marker. 1–8, tomato plants samples; 9, negative control; 10, positive control.

The confirmation that the tomato plants were co-infected with ToCV and TYLCV and its visual details is shown in Figure 1. The non-infected tomato plants did not manifest any symptoms (Figures 1A,B) compared to the symptoms in the co-infected plants (Figure 1E), which had yellowed and curled leaves (Figure 1F). On the contrary, the ToCV-infected tomato plants showed symptoms of chlorosis and yellowing on the leaves (Figure 1I), while the veins were still green (Figure 1J).

The RT-PCR and PCR tests showed that the non-infected plants were not infected with ToCV (Figure 1C) and TYLCV (Figure 1D), respectively. The RT-PCR and PCR tests also confirmed that the tomato plants become co-infected with ToCV and TYLCV after whitefly inoculation, as indicated by the similarity between the target band and the Beijing tomato ToCV isolate (KC887999.1) (Figure 1G) and the Shanghai TYLCV isolates (Figure 1H), which both reached 99%. The target DNA fragment of ToCV was 466 bp, and the target DNA fragment of TYLCV was 1,606 bp. These co-infected plants were used in the subsequent experiments.

For ToCV confirmation, the RT-PCR confirmed that tomato plants were infected with ToCV, but not TYLCV 30 days after whitefly inoculation, as indicated by the similarity between the target band and the Beijing tomato ToCV isolate (KC887999.1) that reached 99.0% (Figure 1K) and the absence of TYLCV (Figure 1L). The target DNA fragment of ToCV was 466 bp. These ToCV-infected plants were used in the subsequent experiments.

### 2.2. Tocv accumulation in *Bemisia tabaci* Med and tomato plants

**Figure 2:**
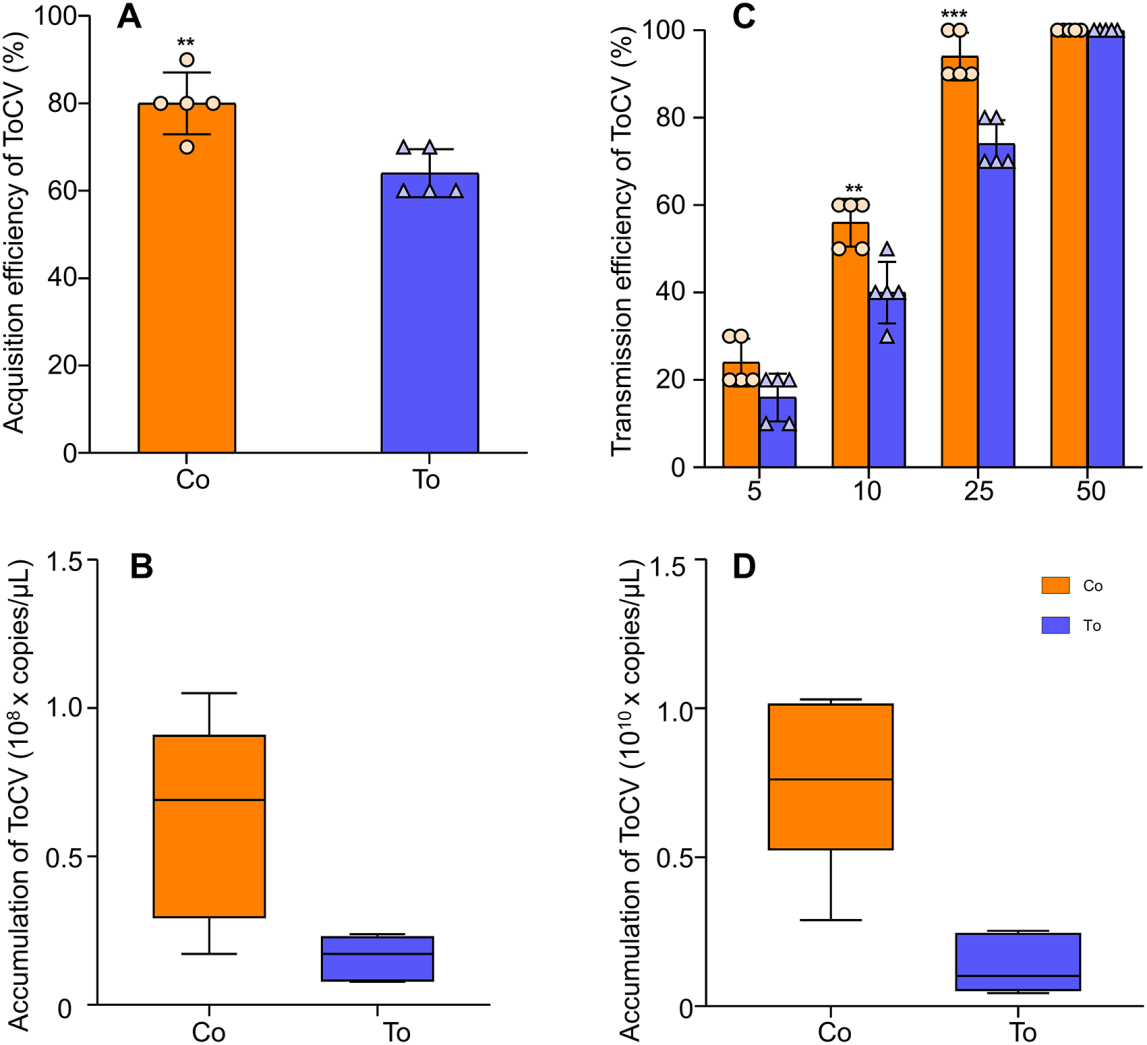
The expression of ToCV and the ability of *Bemisia tabaci* MED to acquire and transmit virus. **(A)** Acquisition rate in *B. tabaci* MED. **(B)** ToCV accumulation in *B. tabaci* MED. **(C)** Transmission rate in *B. tabaci* MED. The horizontal axis shows the number of *B. tabaci* MED to transmit ToCV. **(D)** ToCV accumulation in tomato plants. Co, co-infected *B. tabaci* MED; To, ToCV-infected. Data are denoted as mean ± SE. *** indicates a significant difference at *p* < 0.001; ** indicates a significant difference at *p* < 0.01; * indicates a significant difference at *p* < 0.05.

At 48 h, the acquisition rate of ToCV in *B. tabaci* MED fed on ToCV-infected tomato plants decreased by 20% compared to the acquisition rate of ToCV in *B. tabaci* MED fed on co-infected tomato plants (Figures 2A,F_1,100_ = 4.315, *p* < 0.01). The ToCV accumulation in *B. tabaci* MED fed on ToCV-infected tomato plants after 48 h was 1.59 × 10^7^ copies/μL, and the ToCV accumulation in *B. tabaci* MED fed on co-infected tomato plants was 6.79 × 10^7^ copies/μL, i.e., four times higher than the former (Figure 2B, *F*_1, 30_ = 6.546, *p* < 0.05).

With the different numbers of whiteflies, the transmission rate of ToCV gradually increased. The transmission rate of 10 whiteflies fed on co-infected tomato plants could reach 55%, which was 20% higher than the transmission rate of *B. tabaci* MED fed on ToCV alone. The transmission rate of 25 whiteflies fed on co-infected tomato plants was close to 100%, while 50 whiteflies were needed to reach 100% when fed on ToCV-infected tomato plants (Figure 2C, *F*_1, 10_ = 48.4, *p* < 0.001). ToCV accumulation in tomato plants inoculated with single ToCV-infected whiteflies after 30 days since inoculation was 1.40 × 10^9^ copies/μL, and ToCV accumulation in response to inoculation with co-infected whiteflies reached 7.68 × 10^9^ copies/μL, i.e., approximately five times higher than the former (Figure 2D, *F*_1, 10_ = 20.279, p < 0.01).

### 2.3. Transcriptome sequencing

#### 2.3.1. Quality evaluation of the sequencing

The quality evaluation of the sequencing output data from each sample is shown in Supplementary Table S1. The Q30 value of genes in the combined infected groups and the single infected groups was approximately 94%; the GC content was approximately 40.5%.

#### 2.3.2. DEG analysis

**Figure 3:**
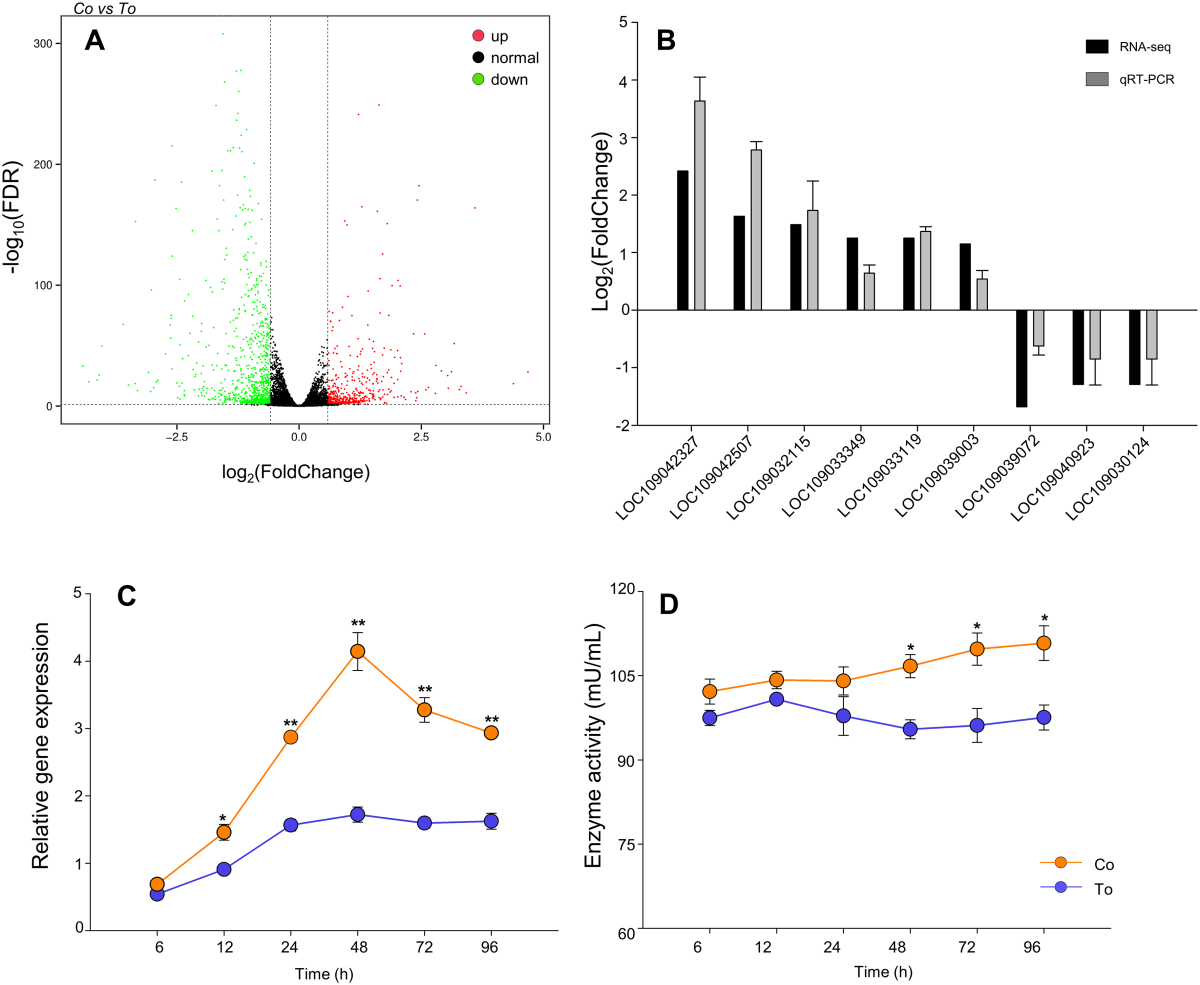
Differentially expressed genes. **(A)** Volcano plot of differentially expressed genes. Red dot shows significantly upregulated genes, green dot shows significantly downregulated genes, black dot represents genes that no significant expression changes, the horizontal axis shows the fold change of gene expression in different samples, and the vertical axis shows the significance of gene expression changes. **(B)** RT-qPCR validation of selected differentially expressed genes. **(C)** The relative gene expression of cathepsin B in *B. tabaci* MED that fed on ToCV and TYLCV co-infected and ToCV-infected tomato plants under various AAP. **(D)** The enzyme activities of cathepsin B in *B. tabaci* MED under various AAP. Data are denoted as mean ± SE. *** indicates a significant difference at *p* < 0.001; ** indicates a significant difference at *p* < 0.01; * indicates a significant difference at *p* < 0.05.

The FPKM distribution of the genes in the co-infection group and the single infection group is shown in Supplementary Figure S1. The DEGs of the two groups were identified by comparing two sets of data. As shown in Figure 3A, a total of 1,410 genes were differentially expressed when whiteflies were introduced to co-infected tomato plants for 48 h AAP, compared to whiteflies on ToCV-infected tomato plants for 48 h AAP, of which 506 were upregulated genes and 904 were downregulated genes.

#### 2.3.3. KEGG analysis

**Figure 4:**
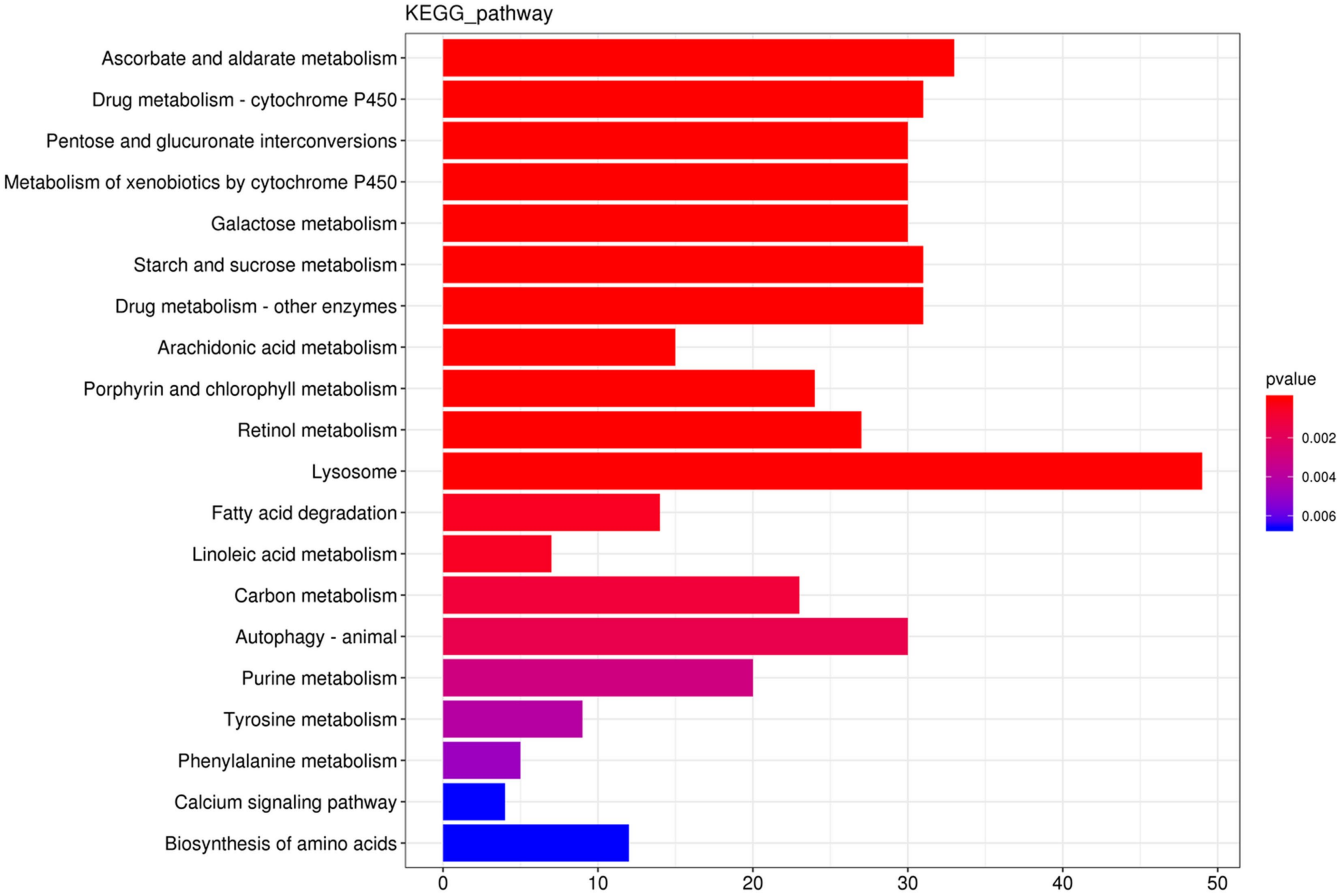
KEGG enrichment diagram. The horizontal coordinate is the gene number, the number of genes of interest annotated in the entry, and the vertical coordinate is each pathway entry. The color of the bars represents the value of *p* of the hypergeometric test.

The results showed that 1,292 DEGs had annotations indicating that these genes belonged to 139 pathways. As shown in Figure 4, the differentially expressed genes between the co-infected and ToCV-infected plants were mainly enriched in pathways involving lysosome metabolism. Compared to whiteflies on ToCV-infected tomato plants, the lysosomal pathway of whiteflies on co-infected tomato plants has the highest number of DEGs (49). Among them, cathepsins in the lysosomal pathway were significantly different.

#### 2.3.4. qRT-RCR verification

The results showed that the expression trend of these genes (LOC109042327, LOC109042507, LOC109032115, LOC109033349, LOC109033119, LOC109039003, LOC109039072, LOC109040923, and LOC109030124) was consistent with the transcriptome results (Figure 3B), which verify the transcriptome sequencing result as credible.

### 2.4. The relative expression and enzyme activity of cathepsin B in *Bemisia tabaci* Med between infection groups

At 6–96 h AAP, the relative expression and enzyme activity of cathepsin B of *B. tabaci* MED fed on co-infected tomato plants increased compared to *B. tabaci* MED fed on ToCV-infected tomato plants. Compared to *B. tabaci* MED fed on ToCV-infected tomato plants, the cathepsin B relative expression of *B. tabaci* MED fed on co-infected tomato plants had a statistical difference for 12, 24, 48, 72, and 96 h AAP. The cathepsin B relative expression of *B. tabaci* MED fed on co-infected tomato plants was 50% higher than that of *B. tabaci* MED fed on ToCV-infected tomato plants for both the AAP of 48 h (Figure 3C, F_1, 28_ = 16.530, *p* < 0.01). The enzyme activity of cathepsin B of *B. tabaci* MED fed on ToCV-infected tomato plants was 20% lower than that of *B. tabaci* MED fed on co-infected tomato plants at 48, 72, and 96 h AAP (Figure 3D, F_1, 28_ = 0.617, *p* < 0.05).

### 2.5. Functional verification of relative gene expression and enzyme activity

#### 2.5.1. Effect of enzyme inhibition treatment on the transmission of ToCV by *Bemisia tabaci* Med

**Figure 5:**
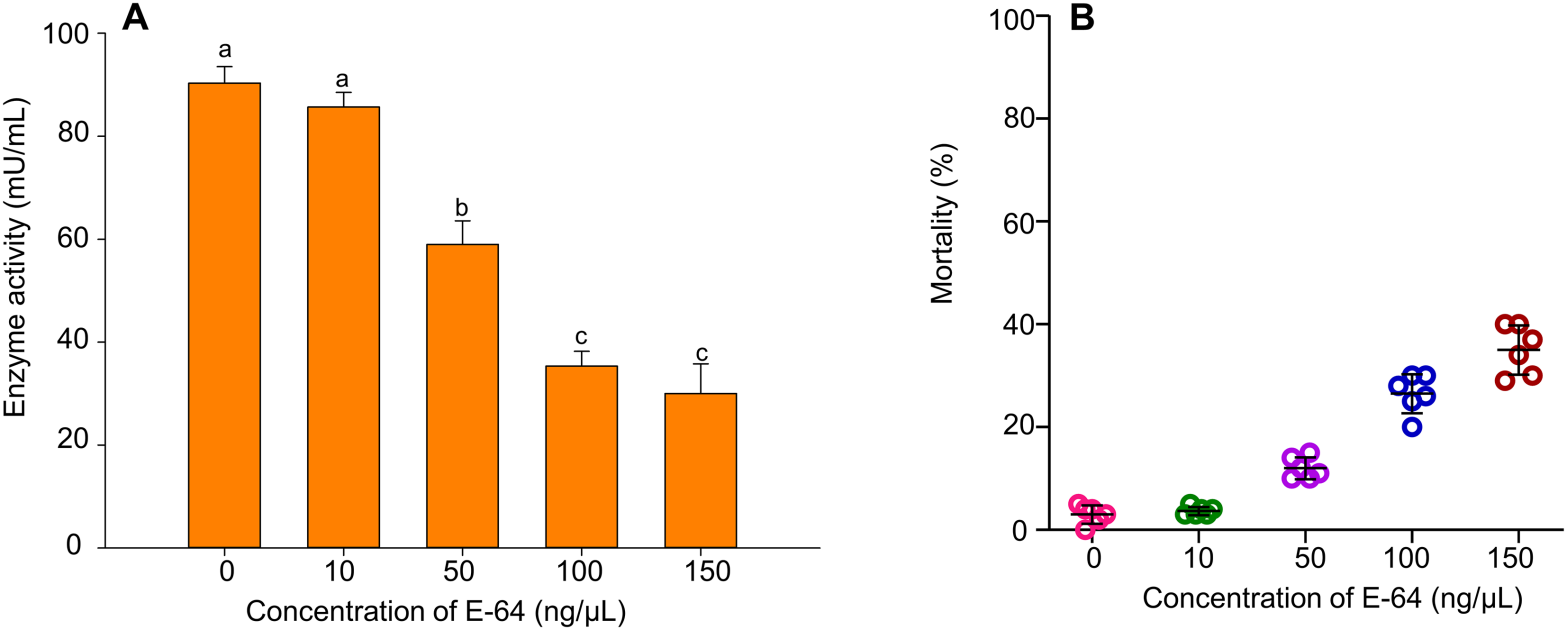
The effect of E-64 treatment on *B. tabaci* MED. **(A)** The enzyme activities of cathepsin B on *B. tabaci* MED of different concentrations of E-64 treatment. **(B)** The mortality effect on *B. tabaci* MED of different concentrations of E-64 treatment. In **(A)**, values are denoted as means±SE; means with different letters are significantly different at *p* < 0.05.

Along with the increase in the enzyme inhibitor Emur-64’s (E-64) concentration, the activity of cathepsin B in *B. tabaci* MED was decreasing while the mortality was increasing gradually. When *B. tabaci* MED was fed a 100 μmol/L feeding solution, the activity of cathepsin B decreased by 50%. (Figure 5A, *F*_1, 28_ = 47.659, *p* < 0.001), the mortality of *B. tabaci* MED could reach 30% (Figure 5B, *F*_4, 100_ = 45.157, p < 0.001).

**Figure 6:**
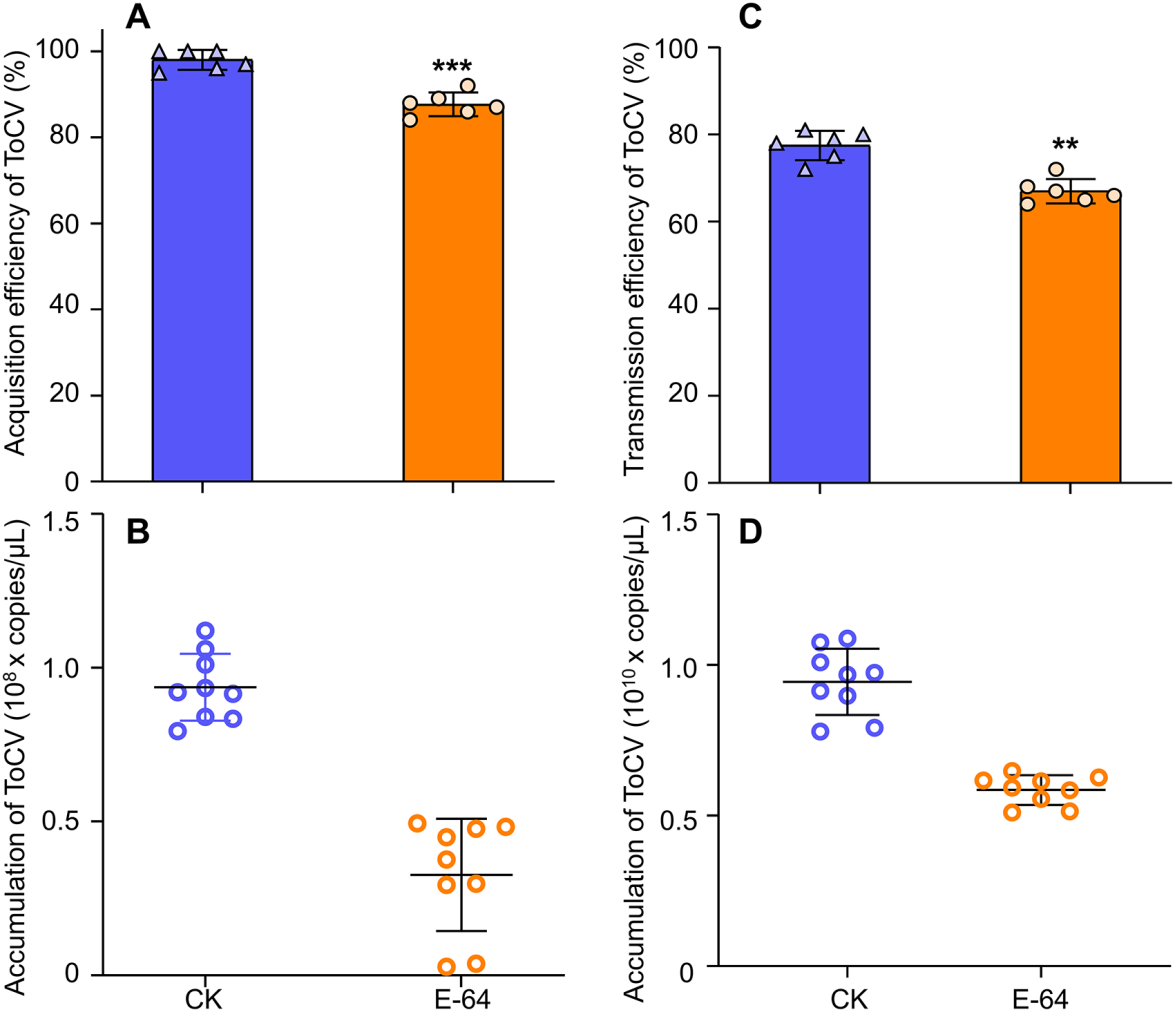
The effects of E-64 treatment on acquisition and transmission in *B. tabaci* MED. **(A)** Acquisition rate of ToCV by *B. tabaci* MED with E-64 treatment. **(B)** ToCV accumulation by *B. tabaci* MED with E-64 treatment. **(C)** Transmission rate of ToCV by *B. tabaci* MED with E-64 treatment. **(D)** ToCV accumulation of in tomato plants exposed to *B. tabaci* MED with E-64 treatment. CK, control; E-64, cathepsin inhibitors. Data are denoted as mean ± SE. *** indicates a significant difference at *p* < 0.001; ** indicates a significant difference at *p* < 0.01; * indicates a significant difference at *p* < 0.05.

The virus acquisition study showed that *B. tabaci* MED that had its cathepsin inhibited had a significantly lower rate of virus acquisition after 48 h AAP than controls, i.e., 15% higher than the former (Figure 6A, *F*_1, 100_ = 0.741, *p* < 0.001). Furthermore, the ToCV acquisition of cathepsin-inhibited *B. tabaci* MED was also lower than that of control with normal cathepsin, i.e., two times higher than the former (Figure 6B, *F*_1, 26_ = 9.036, *p* < 0.01).

For the transmission efficiency of ToCV, the study found that cathepsin-inhibited *B. tabaci* MED had significantly lower transmission efficiency than the control, i.e., 10% higher than the former (Figure 6C, *F*_1, 10_ = 1.000, *p* < 0.01). The ToCV transmission of cathepsin-inhibited *B. tabaci* MED was also lower than that of the control, i.e., approximately two times higher than the former (Figure 6D, F_1, 10_ = 0.293, *p* < 0.001).

#### 2.5.2. Effect of *CathB* dsRNA treatment on the transmission of ToCV by *Bemisia tabaci* Med

The target DNA fragment of cathepsin B was 470 bp, and the target DNA fragment of GFP was 598 bp (Supplementary Figure S2).

**Figure 7:**
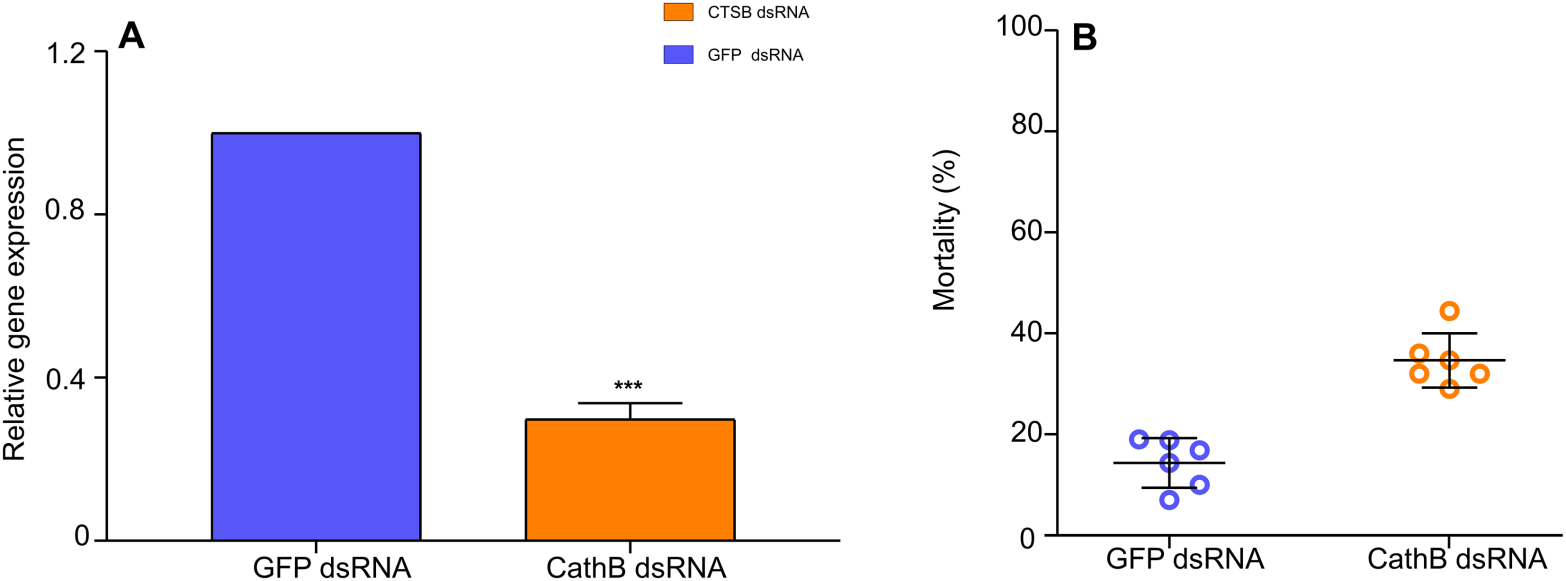
The effect of dsRNAs treatment on *B. tabaci* MED. **(A)** The relative gene expression of cathepsin B on *B. tabaci* MED of dsRNAs treatment. **(B)** The mortality effect on *B. tabaci* MED of dsRNAs treatment. *GFP* dsRNA, control group;*CathB* dsRNA, treatment group. In **(A)**, data are denoted as mean ± SE. *** indicates a significant difference at *p* < 0.001.

After 48 h AAP, compared to 400 ng/μL *GFP* dsRNA in the nutrient solution, the relative gene expression of cathepsin B of *B. tabaci* MED (LOC109042327) decreased by 50% with 400 ng/μL *CathB* dsRNA in the nutrient solution (Figure 7A, *F*_1,31_ = 9.198, *p* < 0.001). *B. tabaci* MED mortality increased with 400 ng/μL *CathB* dsRNA in the nutrient solution (Figure 7B, *F*_1,101_ = 0.014, *p* < 0.001).

**Figure 8:**
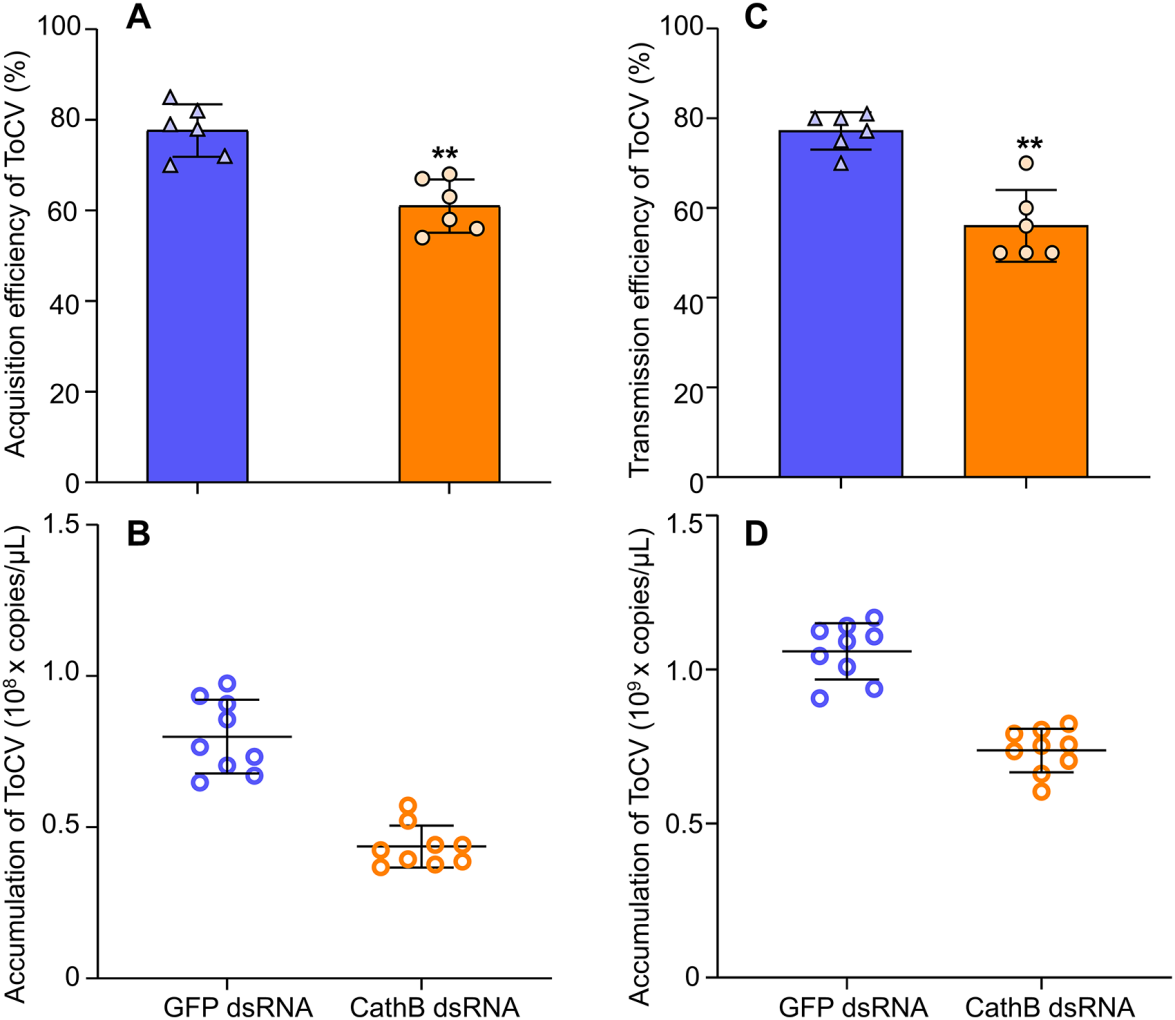
The effects of dsRNAs on acquisition and transmission in *B. tabaci* MED. **(A)** Acquisition rate of ToCV by *B. tabaci* MED with dsRNAs treatment. **(B)** ToCV accumulation of *B. tabaci* MED with dsRNAs treatment. **(C)** Transmission rate of ToCV by *B. tabaci* MED with dsRNAs treatment. **(D)** ToCV accumulation of in tomato plants exposed to *B. tabaci* MED with dsRNAs treatment. *GFP* dsRNA, control group; *CathB* dsRNA, treatment group. Data are denoted as mean ± SE. *** indicates a significant difference at *p* < 0.001; ** indicates a significant difference at *p* < 0.01; * indicates a significant difference at *p* < 0.05.

In comparison to *GFP* dsRNA treatment in the acquisition of ToCV, the acquisition rate of *B. tabaci* MED that was fed with 400 ng/μL *CathB* dsRNA in the nutrient solution was 20% lower (Figure 8A, F_1,101_ = 0.135, *p* < 0.01). Furthermore, the ToCV acquisition of B. tabaci MED fed with 400 ng/μL *CathB* dsRNA was also 50% lower than that of *GFP* dsRNA treatment. (Figure 8B, *F*_1, 28_ = 0.346, *p* < 0.001).

For the transmission efficiency of ToCV, *B. tabaci* MED fed with *CathB* dsRNA had significantly lower transmission efficiency than *GFP* dsRNA, i.e., 20% higher than the former (Figure 8C, *F*_1, 10_ = 1.869, *p* < 0.01). The ToCV transmission of *B. tabaci* MED fed with *CathB* dsRNA was lower than that of *GFP* dsRNA, i.e., approximately 1.5 times higher than the former (Figure 8D, *F*_1, 10_ = 0.868, *p* < 0.001).

## 3. Discussion

TYLCV is a DNA virus with the persistent transmission, while ToCV is an RNA virus with semi-persistent transmission by the whitefly *B. tabaci*. The interaction mechanism between the two different transmission modes of the virus and *B. tabaci* might be different. Through transcriptome analysis of *B. tabaci* MED that fed on co-infected plants from two viruses with different infection modes and a single infection, it was found that when ToCV and TYLCV were present at the same time, the cathepsin B gene of *B. tabaci* MED was significantly upregulated. Meanwhile, we found that the presence of TYLCV promoted the ability of *B. tabaci* to acquire and transmit ToCV with higher efficiency, which indicated that the cathepsin B gene is likely to be one of the key factors regulating the transmission of ToCV by *B. tabaci*.

In recent years, tomato plants showed an upward trend in the co-infection of TYLCV and ToCV (Ding et al., 2019). Potato virus Y acted as an excitation virus to promote the replication of other heterologous viruses (Goodman and Ross, 1974; Pruss et al., 1997). Co-infection of TSWV and ToCV can promote the replication of ToCV in tomato plants, thereby accelerating the death of the host plant (Garcia-eano et al., 2006). We found that there was an increase in the ToCV viral titer and, as a consequence, there was a higher acquisition efficiency of ToCV on co-infected plants than on single-infected plants. Thus, we believe that this is a case of synergistic interaction. Li et al. (2021) found that ToCV and TYLCV mixed-infected tomato plants had a high disease severity index, and ToCV and TYLCV mixed-infected plants with viral accumulation was greater than in singly infected plants (Li et al., 2021). We hypothesize that the ability of whiteflies to acquire and transmit ToCV significantly increased after the co-infection of TYLCV and ToCV by a whitefly protein interacting with ToCV proteins. The silencing suppressor P1/HC-Pro of potato virus A (PVA) significantly increased the accumulation of PLRV in the plant by suppressing the plant defense mechanism after the co-infection of PVA and potato leaf roll virus (PLRV). TYLCV-infected whiteflies can facilitate virus transmission by inhibiting the plant jasmonic acid defense pathway and activating the production of volatile Neophytadiene (Shi et al., 2019a). We hypothesize that TYLCV and ToCV-infected whiteflies could inhibit the defense pathway in tomato plants and thus promote ToCV transmission.

Cathepsins are important enzymes in physiological processes such as protein degradation, cell apoptosis, and signal transduction (Turk et al., 2001; Rossi et al., 2004; Turk et al., 2012), which can regulate the infection and transmission of viruses (Pinheiro et al., 2017). Kaur et al. (2017) found multiple significantly upregulated cathepsin B genes detected by transcriptome sequencing of ToCV-infected *B. tabaci* at 24 h AAP, whereas transcriptome sequencing of TYLCV-infected *B. tabaci* found that multiple cathepsin F and cathepsin B genes were significantly downregulated at 24 h AAP (Hasegawa et al., 2018). The transcriptome sequencing results showed that the cathepsin B gene was upregulated in *B. tabaci* after being fed on the single-infected tomato plants and the co-infected tomato plants. At the same time, the relative expression and enzyme activity of cathepsin B increased after *B. tabaci* was fed on co-infected tomato plants. Therefore, it is speculated that cathepsin B has an inhibitory effect on ToCV in *B. tabaci*.

To date, there are no related reports on cathepsin from *B. tabaci*, but studies on cathepsin from aphids have shown that cathepsin L can regulate the transmission of PLRV by interacting with PLRV on the surface of the intestine in aphids (Pinheiro et al., 2017). PLRV is a persistently transmitted virus that is mainly retained in the midgut of aphids (Terra et al., 2019). ToCV is a semi-persistently transmitted virus and is mainly retained in the foregut of *B. tabaci* (Wang et al., 2019). Studies have shown that cathepsin exists in the salivary glands, saliva, intestines, and honeydew of Hemiptera green bugs and aphids (Lisón et al., 2006; Medina et al., 2015). Cathepsin in *B. tabaci* may have a function similar to that of cathepsin in aphids during the process of ToCV transmission. Gnirß et al. (2012) showed that cathepsin B and L inhibitors activated the Zaire ebolavirus glycoprotein (GP) to reduce 293 T cell infection driven. Cathepsin inhibitors would activate the ToCV CP or CPm protein to reduce ToCV transmission.

The ancestors of Hemiptera insects lost serine peptidase (SP) when they adapted to feed on low-protein plant sap (Terra, 2001; Jongsma and Beekwilder, 2016; Henriques et al., 2017). When they returned to the protein diet, cathepsin was recruited to replace their lost serine peptidase to digest proteins in plant sap (Jongsma and Beekwilder, 2016). Cathepsin B genes were massively amplified in the aphid lineages, and many genes showed intestinal-specific overexpression (Lomate and Bonning, 2016). Therefore, cathepsin not only plays an immune defense function in Hemiptera insects but also acts as a digestive enzyme to digest proteins eaten by insects (Rahbé et al., 2003). After treatment with a cathepsin inhibitor, the activity of cathepsin reduced and digestive enzymes also reduced in whiteflies, resulting in less frequent feeding with tomato plants by whiteflies, thus the ability of *B. tabaci* to acquire and transmit ToCV indirectly decreased.

The expression and activity of cathepsin B in a variety of malignant tissues are significantly higher than in adjacent normal tissues (Fröhlich et al., 2001). Cathepsins play an important role in the invasion of the body by viruses such as Ebola (EBOV) and severe acute respiratory syndrome coronavirus (SARS-CoV); EBOV depended on the activation of cathepsin B and L to invade cells (Gnirß et al., 2012), and SARS-CoV activated the viral envelope protein spines (S) by cathepsin B and L to enter human cells (Simmons et al., 2005). We found that higher expression and activity of cathepsin B lead to an increase in ToCV transmission efficiency. We verified the hypothesis that a decrease in cathepsin B after co-infection could promote ToCV transmission by *B. tabaci*. The silencing of cathepsin B decreased its expression. There may be an antagonistic effect of cathepsin B that activates the entry of viral proteins into *B. tabaci*, thereby facilitating the spread of ToCV.

The interaction among plants, viruses, and insect vectors is very subtle and involves a variety of mechanisms. The studies on *B. tabaci* mainly focus on the characteristics of virus transmission and biology at home and abroad, but there are few studies on the defense mechanism of *B. tabaci*. At present, there are few reports on the interaction between *B. tabaci* of cathepsin and ToCV. Studying the interaction mechanism of cathepsin and ToCV in *B. tabaci* could help to provide new ideas for designing novel control strategies to manage insect vectors and their transmitted viruses.

## 4. Materials and methods

### 4.1. Tomato plants cultivation and whitefly rearing

The tomato variety used was *Solanum lycopersicum* Mill. Cv. Zuanhongmeina, which was cultivated in a greenhouse with an average temperature of 26 ± 1°C, a relative humidity of 70 ± 5%, and a photoperiod of 16:8 l:D cycle. No pesticides were applied to plants, and no other insects were present during plant cultivation other than the introduced *B. tabaci* MED during the experiment. *B. tabaci* MED was originally collected from infested poinsettias (Euphorbia pulcherrima Wild. ex Klotz.) in Beijing, China, in 2009, and was raised in a greenhouse with an average temperature of 26 ± 2°C and a relative humidity of 60 ± 10% (Shi et al., 2019b). The biotypes were identified every 2 months based on the detection of the mitochondrial cytochrome oxidase I gene (*mtCOI*; Supplementary Table S2) based on CAPS (cleavage amplified polymorphic sequence) technology (Chu et al., 2010; Tang et al., 2017). The products were sequenced (Bioengineering Co., Ltd., Shanghai, China), and the results were submitted to the National Center for Biotechnology Information (NCBI) for blast comparison. CAPS-PCR technology and gene sequencing indicated that the insects were *B. tabaci* MED.

The tomato plants were divided into two infection groups, namely, one was co-inoculated with ToCV and TYLCV (co-infected group) and the other was individually inoculated with ToCV (ToCV-infected group). The tomato plants were injected with 0.5 ml of the infectious ToCV cDNA clone or TYLCV DNA clone at the three-true-leaf stage to achieve infection. The infectious cDNA clone of ToCV was provided by Prof. Tao Zhou (China Agricultural University), and the infectious DNA clone of TYLCV was provided by Prof. Xueping Zhou (China Academy of Agricultural Sciences). To verify the presence of the virus, the co-infected and single-infected tomato plants were assayed under PCR with specific primers for TYLCV-F/TYLCV-R and were assayed under RT-PCR with specific primers for ToCV-3F/ToCV-3R (Supplementary Table S2).

### 4.2. Tocv accumulation in adult *Bemisia tabaci* Med females and tomato plants

A minimum of 200 non-infected newly emerged adult whitefly females were starved for 2 h before being transferred to ToCV-infected (100 newly emerged adult *B. tabaci* females) and co-infected tomato plants (100 newly emerged adult *B. tabaci* females) with a similar ToCV virus content and left to feed on the infected plants for 48 h. After 48 h, all *B. tabaci* MED (about 100) was collected, and the acquisition efficiency of ToCV was measured using RT-PCR with specific primers for ToCV-3F/ToCV-3R. At the same time, the accumulation of ToCV on newly emerged adult *B. tabaci* MED females was measured and its ToCV amount was quantified using RT-qPCR by collecting 30 newly emerged adult *B. tabaci* MED females. The total RNA of newly emerged adult *B. tabaci* females was extracted by the TRI reagent (Life Technologies Co., Ltd., Beijing, China). Total RNA was quantified at 500 ng. Hiscript® II Q RT SuperMix for qPCR Kit was used for reverse transcription (Vazyme Biology Co., Ltd., Nanjing, China), and qPCR was conducted by ChamQ Universal SYBR qPCR Master Mix Kit (Vazyme Biology Co., Ltd., Nanjing, China).

On the contrary, to detect ToCV transmission from newly emerged adult *B. tabaci* MED females to tomato plants, 5, 10, 25, and 50 newly emerged adult *B. tabaci* MED females that have fed on infected and co-infected plants were transferred to the non-infected tomato plants that have 3–4 true leaves by using clip-cages. After another 48 h, newly emerged adult *B. tabaci* MED females were removed. The top leaves of tomato plants were collected after 30 days to detect the ToCV transmission and amount. Tomato plants’ total RNA was extracted by the TRI reagent (Life Technologies Co., Ltd., Beijing, China). Total RNA was quantified at 500 ng. Hiscript® II Q RT SuperMix for qPCR Kit was used for reverse transcription (Vazyme Biology Co., Ltd., Nanjing, China); ToCV accumulation in tomato plants was conducted by ChamQ Universal SYBR qPCR Master Mix Kit (Vazyme Biology Co., Ltd., Nanjing, China). For both ToCV accumulation in *B. tabaci* and tomato plants, each treatment was repeated five times.

### 4.3. Transcriptome sequencing analysis

#### 4.3.1. Adult *Bemisia tabaci* Med females virus acquisition and transcriptome sequencing

For virus acquisition, non-infected and newly emerged adult *B. tabaci* MED females were placed in clip-cages (50/cage) and starved for 2 h. The clip-cages were then attached to co-infected plants or ToCV-infected tomato plants for 48 h. This period is known as the acquisition access period (AAP). After 48 h, 300 newly emerged adult *B. tabaci* MED females were collected from infected leaves in an RNA-free centrifuge tube, quickly placed in liquid nitrogen for quick freezing for 30 s, and stored at −80°C. There were three biological replications for each treatment. The six samples were used in transcriptome sequencing, which was performed following the known procedure (Ding et al., 2019; Levin et al., 2020; Servicebio Technology Co. Ltd., Wuhan, China).

#### 4.3.2. Library construction and sequencing

A transcriptome library was constructed using the paired-end RNA-Seq method (Levin et al., 2020). A total of 1 μg RNA per sample was used as input material for the RNA sample preparations. Sequencing libraries were generated using the NEBNext UltraTM RNA Library Prep Kit for Illumina (NEB, USA) following the manufacturer’s recommendations, and index codes were added to attribute sequences to each sample. After the insert size was qualified, the effective concentration of the library was accurately quantified using the RT-qPCR method (effective concentration > 2 nM indicated qualification). According to the requirements of effective concentration and target offline data, after the pooling of different libraries, parametric genome sequencing was performed (reference genome^1^).

To ensure the quality of analysis, it is necessary to filter the original sequence, removing adapters that contained more than 10% N (N indicates that the base information cannot be determined) and low-quality reads (where the base number of *Q*_phred_ ≤ 10 accounts for more than 50% of the total read length). Then, the clean reads can be used for subsequent analysis.

#### 4.3.3. DEG analysis

The edgeR and EBSeq software were used to analyze the gene expression level in each sample. The number of genes with different expression levels and the FPKM value (the expected number of fragments per kilobase of transcript sequence per million base pairs sequenced) of an individual gene was determined. The expression of genes was determined based on FPKM >1.5. The corrected *p*-value (FDR) was used to screen the DEGs (FDR < 0.05). The gene expression levels under different experimental conditions were compared using the FPKM distribution of all genes.

#### 4.3.4. KEGG analysis

To further clarify the biological function and biological pathway of DEGs after *B. tabaci* MED fed on ToCV and TYLCV co-infected tomato plants, the selected DEGs were analyzed by KEGG enrichment using pathway analysis. Through hypergeometric detection, it was found that the DEGs were significantly enriched compared with the whole genome.

#### 4.3.5. RT-qRCR verification

To verify the accuracy of the sequencing results, seven upregulated DEGs and seven downregulated DEGs were screened for RT-qPCR detection to verify the gene expression patterns using RNA-seq data. The TRIzol method (Life Technologies Co., Ltd., Beijing, China) (Fiallo-Olivé et al., 2014) was used to extract the total RNA of 30 co-infected or 30 ToCV-infected *B. tabaci* MED. The uniform quantification was set at 500 ng and the quality of RNA was strictly controlled (A260/280 was set between 1.8 and 2.1). First-strand cDNA was synthesized according to the Hiscript II 1^st^ Strand cDNA Synthesis Kit (Vazyme Biology Co., Ltd., Nanjing, China; Fiallo-Olivé et al., 2014; Pentzold et al., 2017). The primers in the primer sequence (Supplementary Table S2) were used for RT-qPCR detection to obtain the Ct values of each target gene and internal reference genes. Then, the relative expression of each gene was calculated using the 2^-△△Ct^ method (Livak and Schmittgen, 2001). The experiment was repeated three times.

### 4.4. The relative expression and enzyme activity of cathepsin B in adult *Bemisia tabaci* Med females between infection groups

To verify whether the candidate genes are key factors in the lysosomal pathway in regulating the transmission of ToCV by newly emerged adult *B. tabaci* MED females, a significantly upregulated candidate gene was screened: the cathepsin B gene (LOC109042327). The relative expression and enzyme activity of the gene were determined to explore their effects on the transmission of ToCV by adult *B. tabaci* MED females.

Newly emerged adult *B. tabaci* MED females (500) were collected and starved in a clip-cage for 2 h. After that, newly emerged adult *B. tabaci* MED females were transferred to each infection group for an acquisition access period (AAP) of varying duration of 0, 6, 12, 24, 48, 72, and 96 h. After the completion of each duration, 30 newly emerged adult *B. tabaci* MED females were collected from each infection group for the relative expression of cathepsin B was measured. The relative expression of cathepsin B in newly emerged adult *B. tabaci* MED females that acquired the viruses from single-infected and co-infected plants was determined. Fluorescence quantitative PCR detection was carried out by following the ChamQ Universal SYBR qPCR Master Mix Kit instructions (Vazyme Biology Co., Ltd., Nanjing, China). The experiment was repeated three times for each group.

The BCA method was used to determine the protein concentration of newly emerged adult *B. tabaci* MED females by following the BCA protein concentration kit instructions (Solebo Biological Co. Ltd., Beijing, China). The activity of cathepsin B in newly emerged adult *B. tabaci* MED females was determined according to the instructions of the insect cathepsin B ELISA kit (ZCIBIO Technology Co., Ltd., Shanghai, China). The experiment was repeated three times for each group.

### 4.5. Functional verification of relative gene expression and enzyme activity

#### 4.5.1. Effect of enzyme inhibition treatment on the transmission of ToCV by adult *Bemisia tabaci* Med females

Referring to the instructions for Proteinase Inhibitor E-64 (Sigma-Aldrich Trading Co., Ltd., Shanghai, China), cathepsin inhibitor E-64 and 15% sucrose solution (with water as solvent) were prepared into 0, 10, 50, 100, and 150 μmol/L feeding solutions. A total of 50 co-infected newly emerged adult *B. tabaci* MED females (48 AAP) were fed with different concentrations of feeding solution and placed in an incubator with a 14:10 l:D cycle and 80% humidity. Each concentration treatment was repeated three times. After 2 days, the mortality of non-infected *B. tabaci* MED was counted, and the activity of cathepsin B was determined to find the optimal feeding concentration.

To study the effect of enzyme inhibition treatment on the acquisition of virus by newly emerged adult *B. tabaci* MED females, non-infected whiteflies were fed a 20 μL artificial diet solution made of 15% sucrose and 100 μmol/L E-64 cathepsin inhibitor. After a two-day treatment with enzyme inhibition, newly emerged adult *B. tabaci* MED females were collected and starved in a clip-cage for 2 h. After that, newly emerged adult *B. tabaci* MED females were transferred to the co-infection group for a 48-h acquisition access period (AAP). After AAP, all *B. tabaci* MED was collected, and the acquisition efficiency of ToCV was measured using RT-PCR with specific primers for ToCV-3F/ToCV-3R. The experiment was repeated five times. At the same time, 30 *B. tabaci* MED were collected for their ToCV accumulation measured by RT-qPCR. A 20-μL artificial diet that excluded the E-64 cathepsin enzyme inhibitor was used as a control, and the experiment was repeated five times.

To verify the effect of cathepsin inhibition on transmission efficiency, 50 co-infected *B. tabaci* MED that experienced the 48 h AAP were transferred to 3–4 true leaves of non-infected tomato plants in clip-cages, newly emerged adult *B. tabaci* MED were removed 48 h later. After 30 days, the ToCV transmission rate of infected tomato plants was calculated by RT-PCR, and ToCV accumulation of infected tomato plants was measured by RT-qPCR. Tomato plants fed on by newly emerged adult *B. tabaci* MED females previously fed on the artificial diet that excluded E-64 served as the control. Each treatment was repeated five times on each of the 10 tomato plants.

#### 4.5.2. Effect of *CathB* dsRNA treatment on the transmission of ToCV by *Bemisia tabaci* Med

Based on the DNA sequence of the cathepsin B gene (LOC109042327) to clone target DNA fragments, the 470-bp band and the 598-bp band were detected by agarose gel electrophoresis for *CathB* and *GFP*. The *CathB* and *GFP* DNA fragments were subjected to dsRNA synthesis with the T7 RiboMAX Express RNAi system of the dsRNA synthesis kit (PROMEGA, Madison, USA) to obtain *CathB* dsRNAs and *GFP* dsRNAs. In total, 15% sucrose solution (with water as solvent) was prepared into 400 ng/μL *CathB* dsRNA and *GFP* dsRNA feeding solutions. A total of 50 non-infected, newly emerged adult *B. tabaci* MED females were fed with 200 μL of *CathB* dsRNA and *GFP* dsRNA to 400 ng/μL and placed in an incubator with 14:10 l:D cycle and 80% humidity. After 2 days, the mortality of *B. tabaci* MED was counted, and the relative expression of cathepsin B was determined. The experiment was repeated five times.

To study the effect of *CathB* dsRNA treatment on the acquisition of virus by newly emerged adult *B. tabaci* MED females, non-infected whiteflies were fed a 200-μL artificial diet solution made of 15% sucrose and 400 ng/μL *CathB* dsRNA or *GFP* dsRNA. After a 2-day treatment, newly emerged adult *B. tabaci* MED females were collected and starved in a clip-cage for 2 h. After that, newly emerged adult *B. tabaci* MED females were transferred to the co-infection group for 48 h AAP. After AAP, all *B. tabaci* MED was collected, and the acquisition efficiency of ToCV was measured using RT-PCR with specific primers for ToCV-3F/ToCV-3R. The experiment was repeated five times. At the same time, 30 newly emerged adult *B. tabaci* MED females from the co-infection group were collected for their ToCV accumulation measured by RT-qPCR. The experiment was repeated five times.

To verify the effect of *CathB* dsRNA on transmission efficiency, 50 newly emerged adult *B. tabaci* MED females were fed with a 200-μL artificial diet solution made of 15% sucrose and 400 ng/μL *CathB* dsRNA or *GFP* dsRNA at 48 h AAP, then transferred to 3–4 true leaves of non-infected tomato plants in clip-cages, and *B. tabaci* MED was removed 48 h later. After 30 days, the ToCV transmission rate of infected tomato plants was calculated by RT-PCR, and the ToCV accumulation of infected tomato plants was measured by RT-qPCR. Each treatment was repeated five times on each of the 10 tomato plants.

### 4.6. Data analysis

All of the data analyses were performed using SPSS Statistics 21.0 (SPSS Inc., Chicago, IL, USA). Enzyme activity of *AGLU* in whiteflies that fed different concentrations of E-64 was analyzed using a one-way ANOVA followed by Tukey’s test at *p* < 0.05. An independent samples *t*-test was used to compare the acquisition and transmission of ToCV by *B. tabaci*, the relative expression and enzyme activity of cathepsin B, and the effects of *CathB*, *GFP* dsRNA, and E-64 on the acquisition and transmission of ToCV by *B. tabaci*.

## 5. Conclusion

In this study, we verified the hypothesis that the relative expression and enzyme activity of cathepsin B were increased after co-infection, which helped to promote ToCV transmission by *B. tabaci* MED. After the decrease in the activity of cathepsin in *B. tabaci*, its ability to acquire and transmit ToCV was significantly reduced. The silencing of cathepsin B decreased the expression of cathepsin B to reduce the acquisition and transmission of ToCV. This is of profound significance for the prevention and control of diseases and insect pests in the future.

## Data availability statement

The original contributions presented in the study are included in the article/Supplementary material, further inquiries can be directed to the corresponding authors. The raw data supporting the conclusions of this article will be made available by the authors, without undue reservation. Publicly available datasets were analyzed in this study. This data can be found here: NCBI under the accession PRJNA904418.

## Author contributions

X-BS, YL, and D-YZ conceived and designed the experiments. D-Y-HL and J-YL performed the experiments. D-Y-HL analyzed the data. J-BC, YW, Z-HZ, ZZ, L-MZ, and X-QT contributed to reagents/materials/analysis tools. D-Y-HL, X-BS, AF, and X-GZ wrote the article. All authors contributed to the article and approved the submitted version.

## Funding

This study was supported by the National Natural Science Foundation of China (32272535, 32030088, and 31972242), the Agriculture Research System of China (No.CARS-16-E-17 and CARS-23-D-02), and the Support Project of Science and Technology Talent in Hunan Province (2020TJ-Y05).

## Conflict of interest

The authors declare that the research was conducted in the absence of any commercial or financial relationships that could be construed as a potential conflict of interest.

## Publisher’s note

All claims expressed in this article are solely those of the authors and do not necessarily represent those of their affiliated organizations, or those of the publisher, the editors and the reviewers. Any product that may be evaluated in this article, or claim that may be made by its manufacturer, is not guaranteed or endorsed by the publisher.

## Footnotes

^1^ https://ftp.ncbi.nlm.nih.gov/genomes/all/GCF/001/854/935/GCF_001854935.1_ASM185493v1/
